# Convergent methodologies in prosthetic joint infection research: integrating transdisciplinary approaches to understand and prevent biofilm-driven failure of orthopaedic prostheses

**DOI:** 10.1099/jmm.0.002206

**Published:** 2026-09-04

**Authors:** Heather Pinder, Justine K. Rudkin, Nathaniel P.A. Quail, Daniel M. Wall, Peter Young, Liam M. Rooney

**Affiliations:** 1 University Hospital Ayr, NHS Ayrshire & Arran, Ayr, UK; 2 Department of Bacteriology, School of Infection & Immunity, University of Glasgow, Glasgow, UK; 3 School of Medicine, University of Glasgow, Glasgow, UK; 4 Departments of Infectious Disease and Medical Microbiology, NHS Greater Glasgow & Clyde, Glasgow, UK; 5 Centre for the Cellular Microenvironment, University of Glasgow, Glasgow, UK

**Keywords:** cross-scale imaging, host–pathogen interactions, implant-associated infection, multi-omics, prosthetic joint infection, spatial biology

## Abstract

Prosthetic joint infections (PJIs) remain among the most devastating complications of arthroplasty, imposing substantial clinical, economic and patient burdens. Although culture-based diagnostics underpin current clinical practice, PJIs are biofilm-driven infections shaped by taxonomic diversity, spatial organization, host responses and surface interactions, meaning conventional approaches provide only a partial and often decontextualized view of the infection process. We examine how convergent methodologies can transform PJI research by integrating approaches that have traditionally been studied in isolation, including sequencing, transcriptomics, metabolomics, advanced imaging and culture-based characterization. We discuss how whole-genome sequencing, shotgun metagenomics, transcriptomic and metabolomic approaches resolve pathogen identity, functional activity and adaptive persistence and how cross-scale imaging and spatial biology techniques reveal where microbes colonize, interact and survive across implant surfaces. We highlight emerging opportunities to unify these datasets into coherent frameworks that capture both the molecular and physical dimensions of PJIs. Integrating these complementary approaches will enable a multi-layered understanding of PJIs that link composition, function and spatial organization. Ultimately, this provides a foundation for predictive diagnostics, precision antimicrobial strategies and improved implant design and supports a shift towards more effective, mechanism-informed management of implant-associated infection.

## The clinical and epidemiological landscape of prosthetic joint infections

### Epidemiology and clinical burden

Total hip arthroplasty (THA) and total knee arthroplasty (TKA) are among the most frequently performed elective surgical procedures in the United Kingdom, with 116,901 THAs and 134,652 TKAs undertaken in 2024 [1]. Demand is projected to increase by ∼38% by 2060, with the greatest relative increases among patients aged 80 years and over [2]. Prosthetic joint infection (PJI) complicates 1–2% of primary arthroplasties, with higher rates following revision surgery [3]. The cumulative incidence of surgery to treat PJI at 2 years is 3.2 per 1,000 following primary THA and 14.4 per 1,000 following revision TKA [1]. Despite the relatively low incidence, the absolute burden is rising in line with increasing surgical volumes, representing a growing challenge for the National Health Service (NHS) and healthcare systems globally [4].

The economic impact of PJI is disproportionate to its incidence. Recent National Joint Registry (NJR) and Hospital Episode Statistics data demonstrate that patients revised for PJI following primary total hip replacement (THR) in England incurred a mean 5 year inpatient cost of ∼£42,000, compared with £8,000 for matched controls for non-infective cases [5]. PJI also carries a striking mortality risk, with systematic reviews finding a 1 year mortality of ∼4.2–4.3% following two-stage revision for hip and knee PJI, respectively, rising to 21% at 5 years [6, 7]. Importantly, various cohort studies [8–10] show a significantly higher risk of mortality in patients undergoing joint revision surgery for PJI compared to those undergoing the procedure for aseptic revision, matched for potential confounders, such as age and co-morbidities. This data positions PJI not as a surgical complication but as a serious systemic disease with life-threatening consequences.

### Diagnostic challenges and clinical management

Accurate and timely diagnosis of PJI remains challenging, as no single test achieves sufficient sensitivity and specificity in isolation. Several competing classification systems exist, including the Musculoskeletal Infection Society (MSIS) criteria and the 2018 International Consensus Meeting (ICM) scoring system [11]. In Europe, the European Bone and Joint Infection Society (EBJIS) criteria have emerged as the preferred diagnostic framework, employing a stepwise algorithm integrating clinical suspicion, serum inflammatory markers, synovial fluid analysis, microbiological culture and histopathological examination [12]. A multicentre validation study by Sousa *et al*. (2023) confirmed the EBJIS definition as the most sensitive; however, even with agreed diagnostic criteria, the tests these criteria are built on, such as C-reactive protein (CRP), synovial biomarkers like alpha defensin and microbial culture are still lack sensitivity and specificity and the ability to culture the organism [13]. Culture-negative PJI, reported in up to 31% of cases, presents a particular diagnostic challenge [14]. Extended culture protocols, sonication of explanted components and rapid molecular diagnostics, such as the BioFire Joint Infection Panel are increasingly available, though no single innovation has yet supplanted integrated clinical, biochemical and microbiological assessment as advocated by the EBJIS framework [15].

The clinical distinction between acute and chronic PJI – conventionally defined at ∼4 weeks post-surgery or symptom onset [16] – is fundamental to treatment planning. The British Orthopaedic Association (BOA) BOA standards (BOASt) guideline on acute PJI (2023) mandates emergent surgical drainage within 6 h for septic patients and a minimum of five tissue samples for culture, whilst the BOA Specialty Standards (2024) stipulate multidisciplinary team management for all suspected PJI cases [17].

Debridement, antibiotics and implant retention (DAIR) is the first-line surgical strategy for acute PJI with a well-fixed, well-functioning implant and susceptible organism; however, contraindications include sinus tracts, loose implants and resistant organisms [17]. Where DAIR is contraindicated or fails, revision surgery is required. The INFection ORthopaedic Management (INFORM) randomized controlled trial recently established non-inferiority of single-stage (one operation to eliminate infection and reconstruct the joint) compared with two-stage revision (two separate operations, ∼3 months apart), with single-stage associated with £9,450–£10,055 lower costs per patient and comparable re-infection rates in appropriately selected patients [14]. However, interpretation of these findings should consider that INFORM was limited to THAs and was not powered to detect differences in infection recurrence or to stratify outcomes by key biological variables, such as pathogen virulence or polymicrobial infection.

Alongside surgical optimization, antimicrobial strategies are also evolving. The short or long antibiotic regimes in orthopaedics (SOLARIO) multicentre randomized controlled trial [18] has compared short versus long courses of systemic antibiotics in bone and joint infections that are amenable to source control with local antibiotic delivery. Though not yet formally peer-reviewed and published, it purports to show non-inferiority of shorter compared to traditionally longer courses of systemic antibiotics, representing ways in which antimicrobial toxicity-related adverse patient events and general economic burden may be partially mitigated. Nevertheless, with projected annual hospital costs related to hip and knee PJI being estimated to reach $1.85 billion in the USA by 2030 [19], there remains a need for transdisciplinary approaches to understand and prevent this process.

### Why biofilms drive PJIs

The pathogenesis of PJI is fundamentally governed by the capacity of microorganisms to form biofilms on implant surfaces. This process begins within minutes of implantation, when host-derived extracellular matrix proteins, including fibronectin, fibrinogen and collagen, adsorb onto the prosthesis to form a conditioning film. Staphylococcal species, particularly *Staphylococcus aureus* and coagulase-negative staphylococci, exploit this conditioning film through surface-anchored adhesins, initiating irreversible attachment in what was famously described as a ‘*race for the surface*’ between host cells and bacteria [20]. Turner *et al*. recently demonstrated that when *S. aureus* colonizes titanium surfaces before macrophages arrive, biofilm establishment leads to macrophage dysfunction and cell death, with upregulation of biofilm-associated genes and downregulation of the *agr* quorum-sensing system [21].

**Fig. 1.**
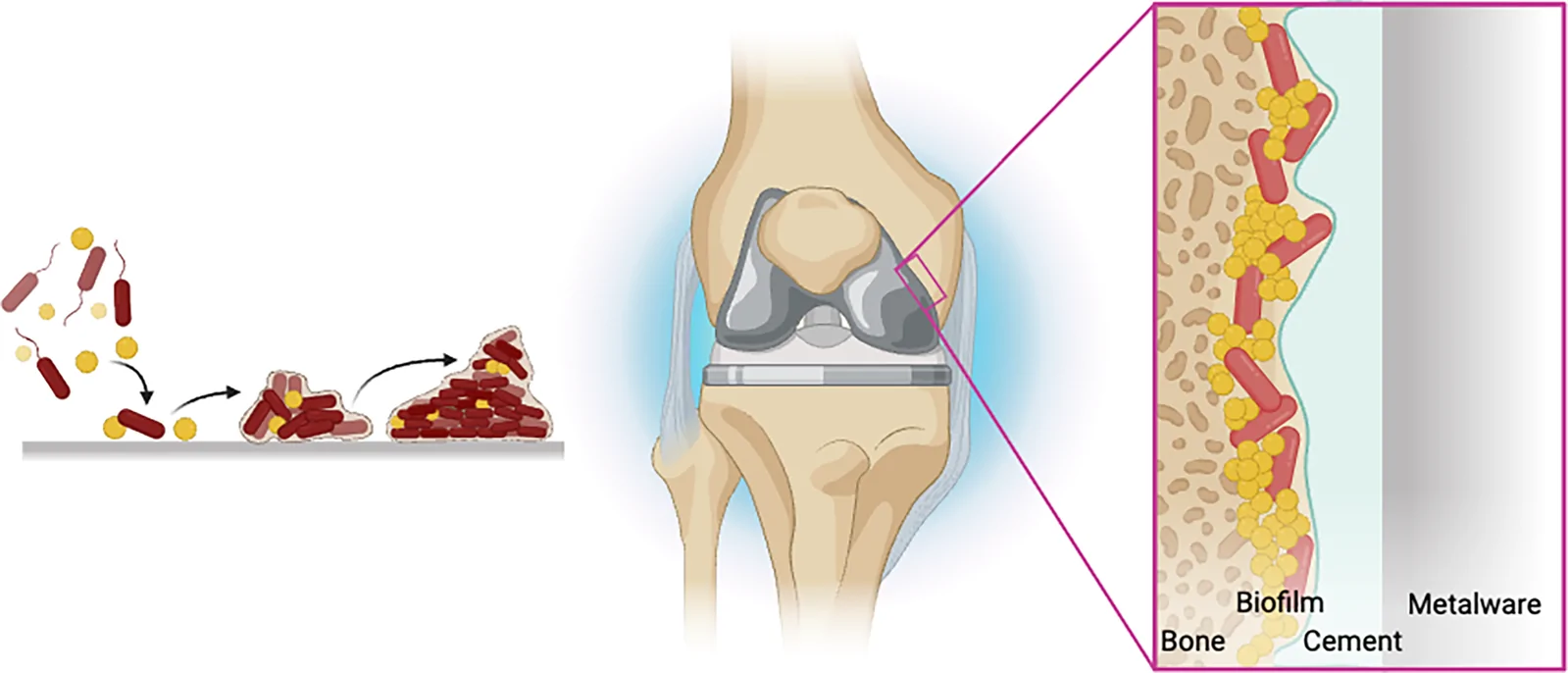
The pathogenesis of PJIs. Biofilm formation underpins the colonization, outgrowth and persistence of PJIs. Free-living cells, originating either from the environment, host flora or contaminated apparatus, colonize abiotic or biotic interfaces and secrete polymeric matrices to embed and protect constituent cells. Three-dimensional growth drives phenotypic and metabolic stratification which creates microniches that promote biofilm tolerance and virulence. PJIs are routinely characterized by periosteal biofilms which establish and grow at the interface of the bone and the prosthesis or adhesive. The expansion and metabolic activity of the biofilm may degrade the bone or prosthesis structures, resulting in loosening of the prosthetic and instability, compounded with the associated inflammatory response to the infection.

Once attached, bacteria transition to a sessile state through production of an extracellular polymeric substance matrix composed of polysaccharides, extracellular DNA and proteins. This colonization pattern can occur anywhere on the implant surface or at the interface of the prosthetic substrate and host tissue (Fig. 1). This mature biofilm confers profound antimicrobial tolerance through restricted antibiotic penetration, metabolic heterogeneity generating persister cell subpopulations and reduced growth rates in deeper biofilm strata [22]. Critically, this tolerance is phenotypic rather than solely genetically encoded, distinguishing it from classical antimicrobial resistance, although both mechanisms frequently co-exist. Biofilm also actively suppresses pro-inflammatory macrophage activity through recruitment of myeloid-derived suppressor cells, polarizing the local immune response towards a tolerogenic phenotype [23]. Furthermore, the generation of small colony variants – metabolically quiescent subpopulations – and intracellular persistence within osteoblasts in certain species provide a reservoir for recurrent infection even after apparent biofilm eradication [24].

## Limitations of traditional diagnostic and experimental approaches for PJI

**Fig. 2.**
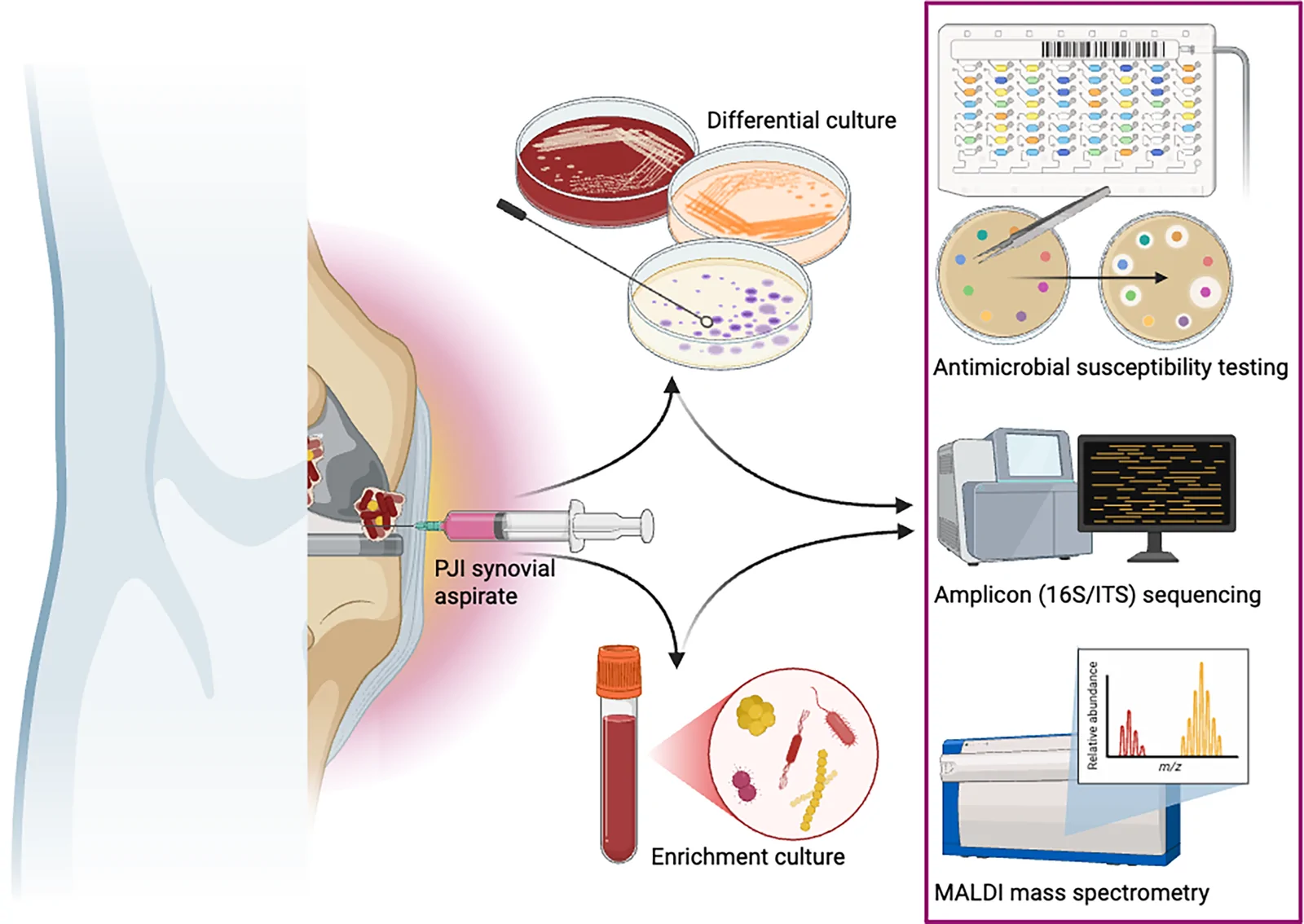
Conventional PJI diagnostic approaches. Routine PJI diagnostics remove the spatial aspect of the infection by either sampling the synovial fluid or debrided tissue in the case of surgical interventions. Patient samples are taken forward for direct isolation on a panel of differential culture media and grown under enrichment conditions to identify more fastidious organisms. PJI isolates are routinely subjected to antimicrobial susceptibility testing and biochemical characterization using the VITEK^®^ platform and amplicon sequencing and MALDI analysis to provide genus-level identification.

Diagnosis of PJIs is complex and often multifactorial. The major criteria for diagnosis are the presence of a sinus co-localized with the prosthesis and/or two separate positive cultures from the affected joint. Diagnosis often takes into account several other additional factors, including: (1) serum biomarkers of infection (CRP, erythrocyte sedimentation rates, IL-6, d-dimer proteins and pro-calcitonin; (2) cytological analysis of synovial fluid (leucocyte counts, polymorphonuclear neutrophil counts; and (3) diagnostic imaging of the affected joint [25, 26]. Identification of the causative organisms is crucial to inform treatment of the infection. Culture-based methods are the current gold standard for achieving this, but these methods are not applied consistently [27]. Culturing also has well-documented limitations, including low sensitivity [28], especially if the patient is receiving antibiotics [29] and biases against fastidious organisms [30]. Polymicrobial infections, which account for 5–37% of all PJIs [31–34], are particularly difficult to identify via culture-based methods, where slow-growing pathogens can be outgrown or outcompeted by faster-growing organisms, masking the presence of fastidious or difficult-to-culture species [35–37]. Diagnosis is further complicated by the presence of biofilms, which are particularly prevalent in chronic PJIs and make recovery of culturable bacteria from the site of infection challenging [38]. Fig. 2 outlines the routine diagnotic approaches used to detect and characterise PJIs.

### The gap between current laboratory models and *in vivo* infection

Infection involves an interaction between the pathogen and the host. The intricacy of such interactions is particularly challenging to replicate within the lab, even prior to considering the additional complexity of polymicrobial infections or the impact of the microbiome. A reliance on culture-based methods limits our ability to research and understand PJIs; by removing the bacteria from the site of infection and culturing them *in vitro*, we lose vital contextual information about the infection.

Biofilms, adherent communities of bacterial cells encased in a protective extracellular matrix, play a significant role in PJIs [39, 40]. In contrast to planktonic cells, bacteria within a biofilm have a lower metabolic rate and are protected from antimicrobial and host immune responses, making infections harder to treat [41]. Biofilms can be visualized on infected tissue and prosthetics [39, 42], but understanding how biofilms form and are maintained *in situ* is crucial for developing effective interventions. Currently, methods to characterize biofilm formation *in vitro* are typically performed on mimetic substrates under laboratory conditions and do not replicate conditions at the site of infection. While these assays have been used to quantify the capacity of PJI-derived bacteria to produce biofilms [43, 44], these approaches do not interrogate the mechanisms of cell-to-surface interactions on materials used in prosthetics. Optimization of experimental conditions to mimic an infection shows that biofilm phenotype is affected by media composition and oxygen concentration, with hypoxic conditions (<2.5% O_2_) and synthetic bone-like environment media increasing biofilm biomass and bacterial cell adhesion to titanium surfaces [45]. Biofilms grown in synthetic synovial fluid also show reduced susceptibility to antibiotics [46], further demonstrating the limitations of *in vitro* assays in PJI research.

### Molecular diagnostics for PJI

In both diagnostics and research, there has been a surge in the use of molecular methods to bypass the limitations of traditional culture. Diagnoses have improved by using 16S and 18S/ITS rRNA PCR, but must be combined with sequencing to confirm causative agents. Whilst DNA sequencing is excellent for confirming which pathogens are present and what genes they possess, it does not inform the metabolic activity or which genes are expressed within the infection context. Whilst explanted bacteria can be cultured and subjected to transcriptome analysis, it is well documented that bacterial gene expression is drastically different within the host compared to growth under lab conditions. For example, strains of Uropathogenic *E. coli* upregulate a core set of genes involved in growth, metabolism and stress response *in vivo*, compared to *in vitro* [47]. The interplay between a pathogen and the host can also affect gene expression, with gut inflammation significantly altering the transcriptome of *Clostridioides difficile* [48]. Strikingly, virulence genes or genes responsible for within-host fitness can vary between different body sites [49] and temporally change over the course of an infection [50]. In the context of PJIs, direct from sample transcriptomics has evidenced the disparity between *in vitro* and *in vivo* gene expression by *S. aureus*, with increased expression of *ica*-independent biofilm genes and genes involved in iron acquisition pathways [51] *in situ*.

In addition to alterations to the transcriptional profile, sonication to dislodge the biofilm from the prosthetic device results in the loss of spatial information. This limits our ability to assess areas of the prosthetic which are vulnerable to biofilm development and destroys information about inter-species interactions and co-localization in polymicrobial biofilms.

## The multi-omics revolution applied to PJI research

The development of high-throughput sequencing technologies has begun to reshape PJI diagnostics. Multi-omics approaches enable interrogation of microbial community composition, gene expression and metabolism, offering new routes to understand complex infections. These methods provide a framework to address three core questions: who is present, what they are doing and how they adapt to persist within the host.

### Whole-genome sequencing and strain-level dynamics

Whole-genome sequencing (WGS) enables high-resolution analysis of individual microbial isolates, providing insight into the genetic relationships and adaptive trajectories of pathogens associated with PJIs. Community-level approaches capture overall composition and activity, but WGS allows direct comparison of isolates across time and between clinical episodes [52, 53]. This is particularly important for distinguishing between persistent infection and reinfection following surgical intervention. The recovery of genetically indistinguishable isolates suggests survival of the original infecting strain, whereas divergent genomes indicate acquisition of a new organism. Distinctions between different emerging pathogens and recurring isolates in PJI relapses have direct implications for clinical management and treatment strategy.

WGS also provides a framework to investigate genetic adaptation under selective pressure, including the acquisition and dissemination of antimicrobial resistance determinants. Mobile genetic elements, such as plasmids and transposons, play a central role in this process, enabling rapid dissemination of resistance and virulence factors within microbial populations [54, 55]. For example, clinical reports of horizontal gene transfer and acquisition of carbapenemase activity and virulence traits have been reported in *Klebsiella pneumoniae* [56] and *Enterococcus faecalis*-driven [57] PJIs, respectively. Additionally, WGS facilitates identification of strain-specific features that influence pathogenic behaviour, including adhesion factors, regulatory systems and resistance mechanisms. These features may contribute to differences in colonization and treatment responses between infections caused by closely related organisms. However, conventional WGS workflows are dependent on successful isolation of organisms, which can introduce biases towards culturable species. While the cost for implementing WGS into diagnostic workflows may be initially high, there is consensus that the real-terms cost saving that WGS imparts outweighs the upfront investment by providing data-rich and one-time assessments of PJI isolates that traditional approaches cannot achieve [58].

### Shotgun metagenomics and community composition

Shotgun metagenomic sequencing provides an unbiased assessment of microbial communities through the analysis of total DNA extracted from biological samples [59]. Unlike targeted amplicon sequencing, which is restricted by primer selection, is limited to genus-level resolution, metagenomics enables species-level identification and, in some cases, strain discrimination [60], while capturing human, bacterial, fungal and viral components simultaneously.

In PJIs, this approach is particularly valuable in identifying organisms that evade conventional culture, including slow-growing, anaerobic or low-abundance taxa. This has important implications for culture-negative infections, where standard diagnostic workflows fail to recover a causative organism. Metagenomic sequencing has, therefore, been explored as an adjunct to routine microbiology, with increasing interest in its application within clinical diagnostic pipelines [61]; for example, in screening of positive PJI blood cultures [62] and in synovial fluid aspirates [63].

Metagenomics also enables characterization of the genetic capacity of microbial communities. Analysis of the resistome of PJI pathogens has identified antimicrobial resistance determinants, while the presence of genes associated with adhesion and immune evasion provides insight into pathogenic potential [64]. However, metagenomic approaches only have the capacity to reflect what organisms *could* do rather than what they *are* actively doing within the infection.

A key limitation is that metagenomics captures a static genomic snapshot, independent of physiological state or environmental context. The presence of resistance or virulence genes does not indicate their expression, nor does it explain how these features contribute to persistence *in vivo*. As such, metagenomics defines who is present and what functions are encoded, but does not resolve active infection processes.

### Metatranscriptomics and functional activity

Metatranscriptomics (or whole-transcriptome sequencing) addresses the functional limitation of metagenomic sequencing by capturing RNA transcripts, providing direct insight into gene expression within microbial communities. In PJIs, metatranscriptomic sequencing has been performed on synovial fluid samples [65], enabling identification of the biological processes that are active during infection, offering a functional perspective that complements genomic data. Others have focused on measuring the host transcriptome of sonicate fluid and cultured samples directly from tissue recovered during PJI arthroplasties, demonstrating conserved and divergent gene expression patterns between staphylococcal and non-staphylococcal PJIs, but they did not discuss the pathogen transcriptome [66]. Other studies have investigated the transcriptome of specific PJI isolates, for example *Streptococcus agalactiae* [67], *Staphylococcus epidermidis* [68] and *S. aureus* [51].

Transcriptional profiling can reveal the activation of stress response pathways, antimicrobial resistance mechanisms [65] and virulence-associated genes [69], reflecting microbial adaptation to host-derived pressures and therapeutic interventions. These include the upregulation of efflux systems [70], quorum sensing [71] and calprotectin-dependent metal tolerance [72]. Such data provide a mechanistic basis for understanding why infections fail to resolve despite appropriate antimicrobial therapy.

However, metatranscriptomic workflows present significant technical challenges. RNA is highly unstable and requires rapid preservation to prevent degradation and transcriptional artefacts [73]. Furthermore, host-derived RNA can dominate sequencing datasets, caused by either low pathogen abundance or ineffective lysis of pathogens compared to host cells. This mandates effective depletion strategies and careful data filtering [74–76].

As with metagenomics, interpretation is constrained by the complexity of mixed-species samples. Despite the challenges surrounding clinical workflow compatibilities, RNA stability and host RNA depletion, metatranscriptomics could provide critical insight into what microbes are doing during infection, linking genetic potential to functional activity.

### Dual RNA-seq and host-microbe interactions

The progression and outcome of PJIs are determined by the interaction between microbial communities and host responses. However, most sequencing-based studies have focused on either pathogen-derived samples or host tissues, but rarely both pathogens and the host within the same analysis. Approaches such as dual RNA sequencing overcome these data siloes by enabling simultaneous analysis of host and microbial transcripts. This provides a means to examine how microbial activity relates to host responses, such as coordinated or opposing transcriptional patterns.

In PJIs, host responses are often characterized by persistent inflammatory processes rather than effective immune clearance of pathogens [77]. Biofilm-associated pathogens can evade immune responses through multiple mechanisms, including modulation of immune signalling pathways and resistance to phagocytic killing [41, 78]. Profiling host gene expression may, therefore, reveal signatures associated with chronic infection, including sustained cytokine production and altered immune regulation [79]. Dual-RNA sequencing may identify co-occurrences of microbial stress-responses and host inflammatory markers which indicate ongoing immune pressure, while reduced host activation may reflect protected niches of infection; both are imperative to understand to provide a complete picture of PJIs.

Despite the ability of these sequencing methods to identify patterns of colonization and activity of complex infections, these approaches remain disconnected from the physical context in which infection occurs. Without linking molecular data to the structural organization of biofilms, interpretation remains incomplete. The integration of multi-omics with spatially resolved imaging is, therefore, required to connect molecular activity with the environments that shape it.

This convergence represents a necessary step towards a more comprehensive understanding of PJIs, in which composition, function and persistence are considered as interconnected components of infection biology.

## The impact of spatial biology and advanced imaging for PJI research

### Imaging biofilms *in situ*

Biofilms have intrinsically complex spatial structure which, in turn, governs the community function, resistance, persistence and colonization patterns. Their structure, function and resilience emerge from the physical organization of microbial cells within a three-dimensional extracellular matrix attached to a surface. Despite this, PJIs have routinely been studied through methods that disrupt spatial context, including sonication, tissue homogenization, joint aspirates and diagnostic culture. While these approaches have provided valuable insight and diagnostic information, they obscure the structural components that underpin biofilm persistence on implant surfaces and PJI interactions with host tissue.

Direct visualization of biofilms *in situ* has, therefore, become an increasingly important objective in PJI research [80]. Early studies relied heavily on scanning electron microscopy (SEM) to demonstrate the presence of microbial aggregates and extracellular matrix on retrieved implants [81–83]. These studies provided compelling visual evidence that biofilm formation occurs on orthopaedic devices ex vivo, confirming long-standing hypotheses derived from *in vitro* work. However, SEM approaches are limited by their relatively small imaging fields and the extensive sample preparation required, which can disrupt biofilm architecture.

Histopathology workflows and routine optical microscopy of orthopaedic specimens have successfully revealed patterns of biofilm colonization across implant surfaces. Various approaches have used absorptive dyes, including the amyloid stain, Congo Red [84], the exopolysaccharide stain, Crystal Violet [85] and the general stain, Methylene Blue [86]. Despite their rapid workflows, these approaches limit the labelling precision of fluorescence-based modalities and can be impacted by spurious binding of organic dyes to implant hardware, potentially creating false positives [87, 88]. However, recent work has shown a dual-sided benefit of using dental plaque disclosing gel as a complementary absorption and fluorescence-based stain for PJI specimens [89].

Confocal laser scanning microscopy (CLSM) enables three-dimensional imaging of biofilms with fluorescent labelling, facilitating visualization of microbial cells embedded within extracellular matrices and quantification of biofilm thickness [90]. CLSM has proven particularly valuable for studying implant-associated infections in experimental models [46] and explanted devices [91]. Fluorescent staining approaches targeting nucleic acids, extracellular polysaccharides and species-specific probes have further enabled differentiation of microbial populations within biofilms. Fluorescence *in situ* hybridisation (FISH) techniques have enabled genus-level differentiation of pathogens which colonize orthopaedic substrates [92] and visualization of transcription events across the surface of substrates would be a powerful approach for PJI research. Other applications of fluorescence imaging have used fluorescent antibody conjugates and dyes to label pathogens or biofilm components directly [93], however, this has yet to be applied to bulk prostheses and at high spatial resolution. A recent advancement for *in situ* fluorescence imaging is the combination of fluorescent antibiotic conjugates and endoscopic imaging to provide diagnostic information at the point of care. In this study, fluorescent vancomycin was applied and imaged using an introspective endoscope device, which facilitated imaging of Gram-positive pathogens in the joint capsule of a PJI [84]. While fluorescent vancomycin would only have revealed the Gram-positive microbes within the PJI niche, recently developed fluorescent polymyxin-B [94] and amphotericin-B conjugates [95] could be coupled with this platform to provide additional visualization of Gram-negative and fungal colonizers, respectively.

Despite these advances, most imaging studies remain restricted to relatively small areas of the implant surface. Orthopaedic implants, particularly knee and hip prostheses, present complex geometries with multiple articulating surfaces, fixation interfaces and microtopographical features [96]. Each of these presents ideal colonization sites for biofilms. Consequently, traditional optical microscopy approaches fail to capture the broader spatial distribution of microbial communities across the entire prosthesis.

Emerging cross-scale imaging platforms aim to bridge this gap by enabling large-area imaging while preserving cellular resolution. Mesoscale optical imaging systems, including the Mesolens [97], have demonstrated the ability to capture millimetre-scale fields of view at subcellular resolution and have proven particularly useful for resolving large populations of single cells within multi-millimetre-sized biofilms [98–102]. These approaches would enable entire implant regions to be mapped while retaining the ability to visualize individual microbial cells. Similarly, light-sheet microscopy techniques would provide powerful multi-scale imaging approaches to scan the surface of implants with high resolution over multiple millimetres [103]. However, these methods would be limited by optical heterogeneity within the specimen that could perturb the quality of the light-sheet illumination. Recent Open Microscopy initiatives may provide a solution to enable low-cost cross-scale imaging of PJIs *in situ*. One of the main challenges is optical access over the complex topology of the implant surface: the EnderScope [104], a 3D printer engineered to repurpose the print head drivers to carry an objective lens, light source and camera housing to provide precise *x*, *y* and *z* control. Adding 3D-printed objective holder capable of pitch, yaw and tilt would provide six-dimensional movement and full angular control of the optics to access normally occluded implant geometries. Advances in 3D printed optics [105–108] and entirely 3D printed microscopes [109] could provide a cost-effective, modular and flexible approach to optical imaging of PJI infections across the implant surface. Cross-scale imaging technologies create new opportunities to examine infection landscapes across implant surfaces rather than isolated biofilm fragments.

This shift towards large-scale imaging is particularly important for understanding treatment failure in PJIs. Clinical evidence suggests that biofilms often persist in protected microenvironments, including implant–bone interfaces and recessed surface features that are inaccessible to surgical debridement [110, 111]. Imaging approaches capable of mapping entire implant surfaces may, therefore, reveal spatial niches that contribute to persistence and recurrence.

### Spatial heterogeneity and microenvironmental structure

The highly organized community structures of biofilms are characterized by steep microenvironmental gradients [112]. Periprosthetic-associated biofilms exhibit gradients in oxygen availability, nutrient concentration and metabolic waste products, even in relatively small aggregates [40]. These gradients create heterogeneous microenvironments that support distinct physiological states within the same microbial community.

Spatial heterogeneity has important implications for antimicrobial tolerance. Cells located in oxygen-limited regions of the biofilm may adopt slow-growing or dormant phenotypes (persisters) that are intrinsically less susceptible to antibiotics targeting active cellular processes [113]. Similarly, the exopolymeric matrices can act as a diffusion barrier, reducing antimicrobial penetration and creating concentration gradients across the biofilm [114]. The combined effect of these spatial gradients creates stratified sub-populations, where the physiological and metabolic profile of pathogens varies, abetting their ability to persist and produce chronic or pervasive infections.

The implant surface itself further contributes to spatial complexity. Orthopaedic prostheses incorporate a range of material surfaces, including polished metal components, porous coatings designed to promote bone integration and cement interfaces [115]. Surface roughness has been shown to vary between different implant substrates, nominally between 150 and 550 nm per 40 µm^2^ [96]. These materials present distinct physicochemical properties, including surface roughness, hydrophobicity and charge [116], all of which influence microbial adhesion and biofilm formation. Moreover, each component can be formulated with antimicrobial agents to either prevent colonization or to treat chronic or low-lying infections [47, 117, 118].

Surface microtopography is particularly important. Micron-scale grooves, pits and manufacturing artefacts can create protected niches that facilitate microbial attachment and shield early biofilm formation from shear forces and host immune responses [119–121]. Over time, these niches may serve as nucleation points from which biofilms expand across the implant surface. Surface micropatterning has also been shown to prevent colonization and spread of pathogens over the surface [122–124]. However, evidence suggests that micropatterned surfaces are easily damaged during implantation and manipulation of the prosthesis [125] or etched in the patient by *in vivo* corrosion [126].

Cross-scale imaging approaches offer the potential to visualize how these physical features interact with microbial colonization patterns. By combining reflection-mode imaging of implant surfaces with fluorescence-based microbial detection, researchers could correlate biofilm distribution with surface morphology. These methods have already proven successful in whole-tissue imaging [127] and would be an impactful addition to the imaging repertoire for PJI studies. These analyses may reveal whether specific implant regions consistently harbour biofilms and, therefore, represent critical targets for prevention strategies.

### Polymicrobial architecture and spatial cooperation

An additional dimension of spatial complexity arises from the polymicrobial nature of many PJIs. While traditional diagnostic approaches often identify a dominant pathogen, increasing evidence suggests that some orthopaedic implant infections can involve multiple microbial species coexisting within structured communities [128–130]. Within biofilms, closely positioned species can engage in metabolic cross-feeding, quorum sensing and cooperative matrix production [131, 132]. Conversely, spatial segregation can facilitate competitive exclusion or antagonistic interactions between neighbouring pathogens.

Imaging studies of polymicrobial biofilms have revealed that different species frequently occupy distinct micro-niches within the same community. For example, facultative anaerobes may colonize oxygen-rich outer regions of the biofilm, while obligate anaerobes persist in deeper layers where oxygen availability is limited [112]. Such spatial partitioning enables diverse microbial populations to coexist within a single infection site and can produce the conditions for antimicrobial tolerance to arise. Some polymicrobial infections also present specific spatial challenges to chemical and surgical interventions. For example, co-infections between *Candida albicans* and *S. aureus* have been documented to increase the recalcitrance and virulence of each pathogen [133, 134]. *Candida* hyphae have also been shown to push staphylococcal cells into micron-scale bone channels [135], establishing chronic osteomyelitis where treatment of indwelling staphylococci is almost impossible. Of course, conversely, antagonistic interactions may suppress some pathogens or alter virulence gene expression.

Understanding the spatial architecture of polymicrobial biofilms, therefore, represents a critical step towards deciphering the ecological dynamics of PJIs. Advanced imaging approaches combined with species-specific probes or FISH techniques may enable the identification of microbial ‘hotspots’ where key interactions occur.

### Linking imaging with spatial ‘omics

While optical and electron microscopy techniques provide structural insight into biofilm organization, it does not directly reveal the functional activity of microbial communities. Integrating spatial imaging with molecular profiling techniques, therefore, represents an important frontier in infection biology.

Recent developments in spatially resolved ‘omics technologies offer promising opportunities to bridge this gap. Techniques such as spatial transcriptomics and imaging mass spectrometry enable mapping of gene expression and metabolite distributions across biological specimens while preserving spatial context. Although these methods have been widely applied in human tissue applications [136, 137], their application to microbial biofilms and implant-associated infections remains in its early stages. This is routinely due to the nature of the probe libraries, which are routinely designed and suited for mammalian cell transcripts. Commercial platforms, such as the 10X Genomics Visium and Xenium workflows, can provide spatial resolution reaching 2 µm, but proprietary probe libraries are currently suited to human and murine tissue samples. A universal 3′ probe library is available but requires poly-adenylated transcripts for efficient capture, which are less common in prokaryotes [138]. Otherwise, if the genetic profile of the pathogens is known and characterized, one may design a custom probe panel for up to ∼500 targets, but this renounces the ability to study complex infections, as are characteristic of PJIs.

Combining cross-scale imaging with spatially resolved molecular analyses could transform our understanding of PJI pathogenesis. For example, imaging data identifying regions of dense microbial colonization could be integrated with transcriptomic profiling to determine which genes are actively expressed within those microenvironments. Similarly, metabolic mapping could reveal the chemical landscape of the infection, including localized nutrient gradients, metabolic by-products or antimicrobial gradients that shape community structure. Imaging mass spectrometry approaches, such as matrix-assisted laser desorption/ionisation - mass spectrometry imaging (MALDI-MSI), have spatially mapped microbial metabolites [139], endotoxin [140] and antimicrobial compounds [141] within tissue sections and have also been adapted for peri-prosthetic tissue and biofilm-associated material [142]. More recently, ambient ionization techniques, such as desorption electrospray ionisation (DESI) mass spectrometry have enabled direct, label-free chemical imaging of biological surfaces under near-native conditions [143], offering potential to analyse irregular or intact implant-associated samples without extensive preparation. In the context of PJIs, DESI-based workflows could be used to map local antibiotic penetration, host-derived inflammatory metabolites or microbial metabolic signatures directly across infected implant surfaces or surrounding tissue. These approaches may also enable the detection of spatially confined metabolic interactions within polymicrobial communities, such as cross-feeding or the local accumulation of inhibitory compounds, which are not resolvable through bulk metabolomics. Challenges remain in achieving sufficient spatial resolution for microscale biofilm structures, as well as in disentangling overlapping host and microbial metabolite signatures. However, recent advances in low-flow and nano-DESI approaches have achieved spatial resolution at 5–10 μm [144].

Ultimately, the convergence of spatial imaging and molecular profiling may enable the construction of multi-layered maps of implant infections that integrate structural, genetic and functional information. Such datasets would provide unprecedented insight into how pathogens colonize, organize and persist on orthopaedic implants. As spatial biology technologies continue to mature, their application to PJIs is likely to accelerate. Integrating these approaches with advances in multi-omics sequencing and implant engineering may facilitate a more comprehensive understanding of infection ecology, providing the foundation for new diagnostic tools and preventive strategies.

Table 1 outlines the outputs, strengths and limitations, clinical readiness, and challenges of implementing each of the methods discussed above into PJI research.

**Table 1.** Comparative summary of emerging and established methodologies for PJI research and translation

*Approach*	*Primary output*	*Key strength*	*Key limitation*	*Clinical readiness*	*Implementation challenge*
***Culture and antimicrobial susceptibility testing***	Viable isolates and susceptibility profiles	Clinically embedded Enables isolate banking	Low sensitivity Culture bias Loss of spatial context	Established	Standardization across clinical sites Detection and isolation of polymicrobial infections
***Sonication***	Biofilm dissociation	Improves pathogen recovery	Destroys spatial context	Established	Standardization of protocols and instrumentation
***WGS***	Strain-level genomes	Ability to track persistent or reinfecting isolates Detection of AMR and virulence genes	Requires culture	Increasing	Cost Expertise Turnaround time in context of clinical decision making
***Shotgun metagenomics***	Phylogenetic profiling Resistome profiling Metagenome-assembled genomes (MAGs)	Culture independent Suitable for profiling polymicrobial communities	High abundance of host DNA can outweigh pathogen DNA or cause contamination Can require specialist interpretation and analysis	Emerging	Bioinformatics demand and training Detection thresholds for low-abundance pathogens or to discern pathogens from host commensal contamination
***Metatranscriptomics***	Active gene expression	Functional activity	RNA instability and breakdown Requirement to deplete host RNA	Experimental	Requires rapid and robust sample handling, extraction and processing Cost
***Metabolomics and Mass Spectrometry Imaging***	Metabolite and antibiotic distributions	Chemical activity Antibiotic gradients	Annotation Resolution Difficulty in interpreting mixed host and microbe signals	Experimental	Cost Specialist instrumentation Requirement for comparative standards
***FISH and other advanced imaging methods***	Spatial localization of microbes Pathogen phenotyping	Direct visualization	Limitation between field of view and resolution with conventional microscopes Labelling constraints and specific fluorescent labelling of pathogens versus host	Experimental	Probe design requires a priori knowledge of pathogen(s) Throughput Limited commercial fluorescent dyes specifically for microbes
***Cross-scale imaging***	Prosthesis-scale spatial visualization of infection	Large capture volume with cellular context	Access to instrumentation Specialist training and tools Data volume and handling	Experimental	Specialist hardware, instrumentation and training High-performance computing resource for multi-scale analyses
***Artificial intelligence/machine learning integration***	Prediction and classification of infections	Pattern detection Risk models	Can be impacted by training biases Can be challenging to interpret and validate outputs	Emerging	Harmonized datasets External validation

MAGs, metagenome-assembled genomes.

## A convergent approach to understanding PJI pathogenesis

Combining multi-omics and spatial imaging approaches provides a foundation to move beyond descriptive microbiology towards a systems-level understanding of PJIs. Rather than viewing infections as collections of isolated pathogens, these approaches enable interrogation of PJIs as structured, dynamic systems, in which microbial composition, functional activity, spatial organization and host responses are intrinsically linked.

Integrating normally siloed approaches creates the potential to develop predictive models of infection behaviour. Advances in computational biology and artificial intelligence (AI) provide a framework to analyse high-dimensional datasets derived from sequencing and imaging workflows, enabling identification of patterns that are not apparent through conventional analysis. Such approaches could allow inference of microbial interactions, identification of persistence-associated phenotypes and prediction of treatment response based on the combined structural and molecular features of infection. In this context, AI provides a mechanism to integrate disparate datasets into coherent models to better understand infection dynamics.

Convergence of cross-disciplinary approaches supports the emergence of precision-informed therapeutic strategies. By linking microbial identity, gene expression and functional state to infection outcomes, it becomes possible to rationalize treatment selection beyond empirical antimicrobial use. Therapeutic approaches may, therefore, be guided by the specific characteristics of the infection, including resistance profiles, metabolic activity and host-microbe interactions. Such strategies have the potential to move PJI management towards targeted, mechanism-informed interventions.

**Fig. 3.**
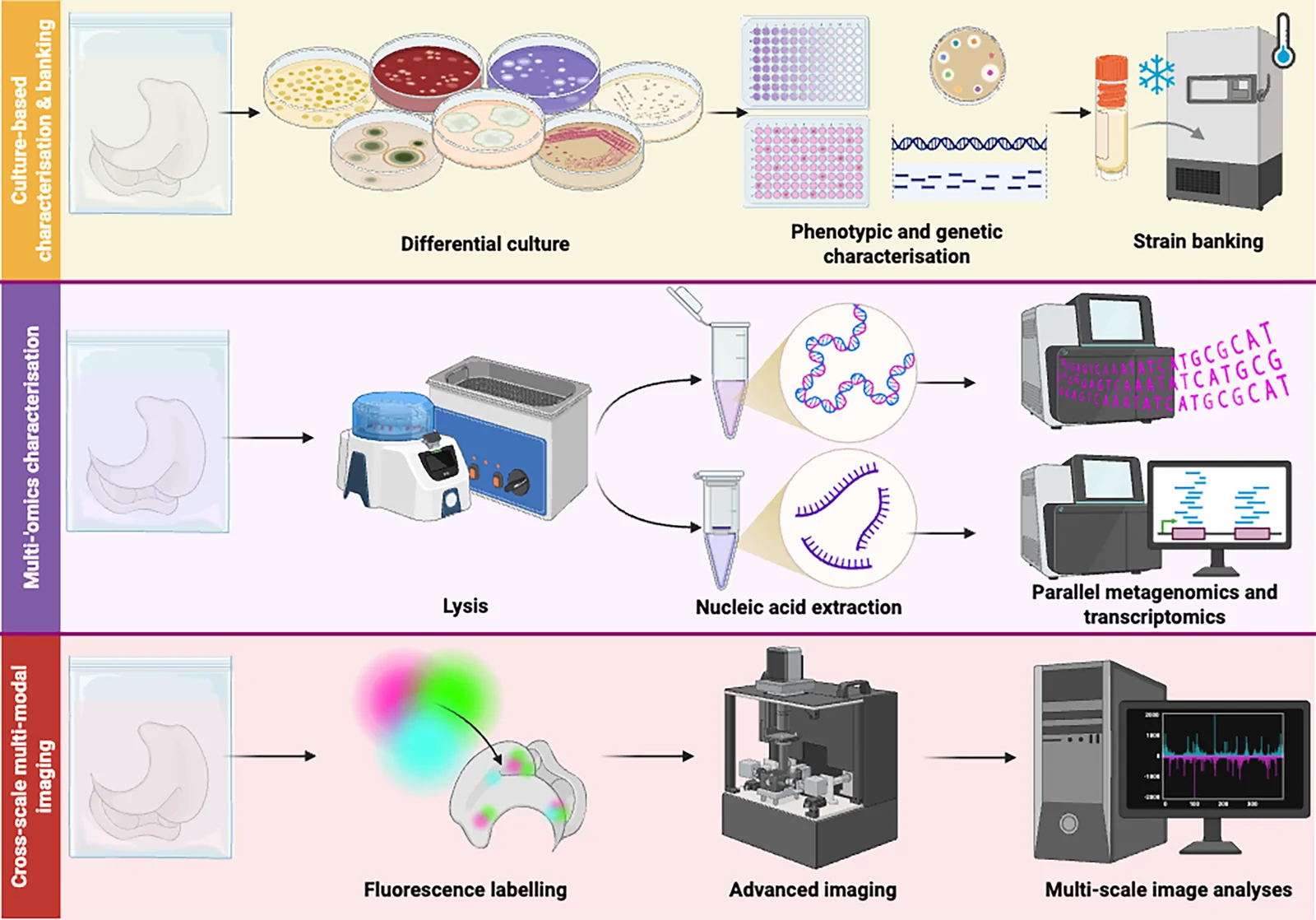
A proposed convergent workflow for PJI research. Three parallel workflows are outlined which provide multi-variate information to elucidate PJI infections and decode their makeup, effect on the host and spatial distribution. The conventional healthcare diagnostic workflows for differential culture are followed by characterization of biofilm formation, virulence and antimicrobial susceptibility and identification and gene carriage are confirmed by WGS. PJI isolate banking would provide a vital resource for PJI research and secondary studies. These culture-based approaches could be supported by parallel metagenomics and whole transcriptome sequencing, providing taxonomic and functional information in direct context of the infection. Finally, incorporating cross-scale multi-modal imaging would provide key spatial information for PJI researchers and prosthesis manufacturers, identifying hotspots of infection across the implant surface.

However, realizing this vision requires a transition in how PJI research is conceptualized and conducted. Multi-omics and imaging approaches generate datasets that are inherently complex and interdependent, requiring integration across methodological and disciplinary boundaries. Fig. 3 describes a conceptual framework that combines these approaches to better understand and provide secondary resources for PJI research. Bridging these divides will be essential to ensure that advances in molecular microbiology, imaging and computational analysis translate into clinically meaningful insight.

A systems-level model of PJI pathogenesis, therefore, represents both a scientific and structural shift. By integrating spatial, molecular and computational perspectives, it becomes possible to transition from static descriptions of infection towards predictive, mechanistic understanding, providing a framework for the development of more effective diagnostic and therapeutic strategies.

## The future of PJI research: AI, precision therapeutics and implementation

### AI in clinical risk prediction, decision-making and stratification

AI and machine learning (ML) approaches are increasingly being explored as tools to support predictive diagnostics, risk stratification and clinical decision-making in PJI. Supervised models trained on electronic health record data have shown potential in preoperative PJI risk stratification, enabling personalized risk assessment and targeted prophylactic intervention [145, 146]. Such tools could support shared decision-making by helping patients and clinicians contextualize individual risk before arthroplasty, particularly in patients with multiple comorbidities or previous revision surgery.

Beyond diagnosis, AI may also support therapeutic decision-making by integrating patient-level risk factors, microbiological data, antimicrobial susceptibility profiles, surgical history and longitudinal outcomes [147]. Such approaches have already begun predicting recurrence risk, identifying patients most likely to benefit from DAIR versus revision surgery or informing antimicrobial treatment regimes [148–150]. However, these applications remain largely conceptual in the context of clinical PJI management and will require robust prospective validation before widespread deployment.

### AI in clinical microbiology and laboratory diagnostics

AI also has potential to support clinical microbiology and laboratory diagnostics, particularly as molecular and omics-based approaches generate increasingly complex datasets. In principle, ML models could assist with pathogen classification [151], antimicrobial resistance prediction [152], clinical data cleaning [152], interpretation of polymicrobial signatures [153] and prioritization of clinically relevant organisms from metagenomic or metatranscriptomic datasets [154]. These applications may be particularly valuable in culture-negative or diagnostically ambiguous PJI, where conventional microbiology provides incomplete, inconclusive or conflicting results. However, sampling bias can confound AI and ML approaches, which has been reported to impact clinically important predictions, such as antimicrobial resistance (AMR) [155].

### AI in radiology, imaging and biofilm analysis

Imaging represents another major area in which AI will accelerate PJI research and translation. Kampik *et al*. recently demonstrated that visible and near-infrared hyperspectral imaging coupled with ML classifiers achieved classification accuracies of up to 99.6% for distinguishing infected from uninfected human bone samples ex vivo, with the capacity to differentiate between *S. aureus* and *S. epidermidis*, offering a potential real-time, label-free intraoperative diagnostic tool [156]. Deep learning-based clinical classification systems for radiographic differential diagnosis of prosthetic failures have also been validated in multicentre studies [157]. In parallel, AI-driven approaches are being applied to biofilm biology itself: deep temporal sequence classification algorithms now enable automated single-cell tracking within dense three-dimensional biofilm microscopy datasets, offering unprecedented resolution of spatial heterogeneity and population dynamics [158].

### Precision antimicrobial and anti-biofilm therapies

AI-driven therapeutic design is also emerging, for example in phage therapy design. The concept of ‘smart phage’, where AI is used to predict phage-host interactions, optimize phage cocktail selection and model resistance emergence, represents an emerging possibility for guided precision therapeutics [159]. Structured compassionate-use programmes, such as PHAGEinLYON, are currently providing clinical access frameworks, albeit with regulatory challenges surrounding pharmaceutical-grade phage production and personalized matching [160, 161]. Nanoconjugate delivery systems, including phage-liposome constructs, have demonstrated enhanced biofilm penetration and eradication of multidrug-resistant pathogens in orthopaedic models [162].

Immunotherapeutic approaches are equally promising: TRL1068, a human monoclonal antibody targeting a conserved biofilm-anchoring protein (DNABII), has restored antibiotic susceptibility in orthopaedic implant infection models, where scanning electron microscopy has confirmed disruption of three-dimensional staphylococcal biofilms on treated implants [163]. These approaches are complemented by novel antibiotic-independent strategies, including ClpP-activating antibiotics that induce bacterial protein degradation within biofilms, electromagnetic induction heating of metallic implants to physically disrupt biofilm integrity and programmable drug delivery platforms utilizing electrochemical corrosion of liquid metal nanoparticles for precision-controlled release [164, 165]. Collectively, these technologies signal a shift towards mechanism-informed, pathogen-specific treatments.

### Regulatory, analytical and implementation barriers

Despite advances in AI approaches to microbiology and clinical practice, significant regulatory and implementation barriers remain [166]. The translation of AI diagnostic tools into clinical practice requires prospective validation across diverse patient populations, standardization of data inputs and integration into existing clinical workflows [167]. These challenges are amplified by the heterogeneity of PJI presentation and the absence of universally accepted diagnostic gold standards [168]. At present, the gold standard for diagnosis remains the presence of a sinus tract leading to the implant or positive culture of the same organism on two separate aspirates of joint fluid. Whilst other biomarkers may strengthen the diagnosis, there remains a paucity of highly specific and sensitive imaging or laboratory diagnostic tests. Models trained on retrospective, single-centre or highly selected datasets may perform poorly when applied to larger cohorts, particularly where diagnostic criteria, coding practices, microbiology workflows, imaging protocols or surgical pathways differ.

Data harmonization is essential to standardize and validate the implementation of AI to clinical workflows. AI and multi-omics approaches require standardized clinical metadata, sample processing, sequencing pipelines, imaging acquisition protocols, annotation and outcome definitions. Model interpretability is equally important in clinical settings, where black-box predictions may be difficult to act upon without mechanistic explanation or clear uncertainty estimates. Bias also represents a major concern, as models may encode differences in access to arthroplasty, referral pathways, comorbidity burden, ethnicity, socioeconomic status or healthcare utilization [169, 170]. External validation, prospective evaluation and clinically meaningful endpoints will be required before AI-enabled PJI tools can support routine diagnosis and patient management.

Bacteriophage therapy faces particularly complex regulatory hurdles. Current pharmaceutical licensing frameworks, designed for standardized chemical entities, are poorly suited to the personalized and biologically variable nature of phage-based treatments, necessitating regulatory pathways that balance safety, efficacy and quality control [160]. Furthermore, the multi-omics and spatial biology approaches discussed throughout this review generate datasets of extraordinary complexity, requiring robust bioinformatics infrastructure, standardized analytical pipelines and interdisciplinary expertise that is not yet routinely available within clinical microbiology or orthopaedic research settings [171]. The cost of this infrastructure may be initially prohibitive, particularly in health services, such as the NHS, unless supported through coordinated research networks, shared analytical platforms and reciprocal training.

### Building a transdisciplinary PJI research community

Realizing the promise of convergent PJI research will ultimately require avoidance of disciplinary silos that have historically fragmented broad fields of research, such as PJI. Progress demands structured collaboration between clinical microbiologists, orthopaedic surgeons, biofilm biologists, materials scientists, bioinformaticians, immunologists and patients. This is particularly important because no single methodological approach can fully resolve the biological, spatial and clinical complexity of PJI.

In the UK, recent funding initiatives reflect growing recognition of this need. The UK Research and Innovation (UKRI) Transdisciplinary Antimicrobial Resistance programme has invested £4.8 million in Phase 1 networks to connect and expand cross-sector AMR research communities, with Phase 2 awarding up to £15 million for larger-scale transdisciplinary research projects, co-funded by NIHR and DEFRA [172]. The NIHR has additionally established a Translational Research Collaboration in Infection and Antimicrobial Resistance, hosted at Imperial College London, to coordinate experimental vaccinology, diagnostic standardization and strategic treatment studies [173]. At the European level, Horizon Europe 2026–2027 work programmes and the [174] on new treatments to tackle AMR through the One Health AMR Partnership provide further funding architectures for cross-border, multidisciplinary infection research [174]. These initiatives could embed PJI within broader infection and AMR research ecosystems, fostering data-sharing frameworks, multi-centre biofilm imaging registries, linked isolate and metadata repositories and translational pipelines that bridge the gap from spatial and molecular characterization to patient-facing interventions.

## Conclusion

PJIs remain difficult to treat, diagnose and prevent because they are biologically complex, spatially structured and shaped by interactions between pathogens, host tissues and implant surfaces. Culture, genomics, transcriptomics, metabolomics, imaging and phenotypic assays each provide useful, but incomplete, perspectives. Their greatest value lies in their potential to integrate and create a complementary approach that is reproducible, interpretable and clinically meaningful. A convergent PJI research framework should connect microbial identity with functional activity, spatial organization, biofilm phenotype, antimicrobial susceptibility and host response. Such integration would support improved risk stratification, more precise therapies, better-informed prosthesis design and more robust models for infection recurrence and management. However, achieving this will require standardized sampling, shared isolate and imaging resources, validated analytical pipelines and sustained collaborations across clinical and research disciplines. By moving from siloed methods towards systems-level understandings of implant-associated infection, the field can better define where pathogens persist, how PJI biofilms tolerate treatment and why infection recurs. This shift will be essential for translating mechanistic insight into predictive, personalized and mechanism-informed PJI diagnosis, prevention and management.

## Abbreviations

AI: artificial intelligence
AMR: antimicrobial resistance
BOA: British Orthopaedic Association
BOASt: BOA standards
CLSM: confocal laser scanning microscopy
CRP: C-reactive protein
DAIR: debridement, antibiotics and implant retention
Defra: Department for Environment, Food and Rural Affairs
DESI: desorption electrospray ionisation
EBJIS: European Bone and Joint Infection Society
FISH: fluorescence *in situ* hybridisation
ICM: international consensus meeting
INFORM: infection orthopaedic management
MALDI-MSI: matrix-assisted laser desorption/ionisation mass spectrometry imaging
ML: machine learning
MSIS: Musculoskeletal Infection Society
NHS: National Health Service
NIHR: National Institute for Health and Care Research
NJR: National Joint Registry
PJI: prosthetic joint infection
SEM: scanning electron microscopy
SOLARIO: short or long antibiotic regimes in orthopaedics
THA: total hip arthroplasty
THR: total hip replacement
TKA: total knee arthroplasty
UKRI: UK Research and Innovation
WGS: whole-genome sequencing
